# Toll-Like Receptor 9 Deficiency Protects Mice against *Pseudomonas aeruginosa* Lung Infection

**DOI:** 10.1371/journal.pone.0090466

**Published:** 2014-03-04

**Authors:** Fatima BenMohamed, Mathieu Medina, Yong-Zheng Wu, Sophia Maschalidi, Gregory Jouvion, Laurent Guillemot, Michel Chignard, Bénédicte Manoury, Lhousseine Touqui

**Affiliations:** 1 Unité de Défense Innée et Inflammation, Institut Pasteur, Paris, France; 2 INSERM, U.874, Paris, France; 3 INSERM, U.1013, Paris, France; 4 Unité d'histopathologie humaine et modèles animaux, Institut Pasteur, Paris, France; Louisiana State University, United States of America

## Abstract

*Pseudomonas aeruginosa* is an opportunistic pathogen involved in nosocomial infections. While a number of studies have demonstrated the roles of TLR2, TLR4 and TLR5 in host defense againt *P. aeruginosa* infection, the implication of TLR9 in this process has been overlooked. Here, we show that *P. aeruginosa* DNA stimulates the inflammatory response through TLR9 pathway in both a cell line and primary alveolar macrophages (AMs). This activation requires asparagine endopeptidase- and endosomal acidification. Interestingly, TLR9^-/-^ mice resisted to lethal lung infection by *P. aeruginosa*, compared to WT C57BL/6 mice. The resistance of TLR9^-/-^ mice to *P. aeruginosa* infection was associated with: (*i*) a higher ability of TLR9^-/-^ AMs to kill *P. aeruginosa*; (*ii*) a rapid increase in the pro-inflammatory cytokines such as TNFα, IL-1β and IL-6 production; and (*iii*) an increase in nitric oxide (NO) production and inductible NO synthase expression in AMs. In addition, inhibition of both IL-1β and NO production resulted in a significant decrease of *P. aeruginosa* clearance by AMs. Altogether these results indicate that TLR9 plays a detrimental role in pulmonary host defense toward *P. aeruginosa* by reducing the AMs clearance activity and production of IL-1β and NO necessary for bacteria killing.

## Introduction

Pulmonary infections represent a major cause of mortality by infection in the world [1]. In immuno-compromised patients, exposition of respiratory tract to infectious pathogens results in serious and life-threatening diseases [2]. *Pseudomonas aeruginosa*, a Gram-negative bacteria, is an important cause of pulmonary infections in immuno-compromised patients and in patients with cystic fibrosis [3], [4].

The ability of the host to respond to invading pathogens is in part attributed to a family of receptors called toll-like receptors (TLRs), which recognize conserved pathogen associated molecular patterns (PAMPs) present in microbes [5]. TLRs-PAMPs interactions initiate innate immune defence against pulmonary pathogens [5]. Previous studies have demonstrated the protective role of TLR2, TLR4 and TLR5 in host defence following lung infection with *P. aeruginosa* [6], [7], [8], [9]. However, whether or not TLR9 plays a protective or deleterious role in host defence against *P. aeruginosa* remained to be determined. TLR9 plays an essential role in activating innate immunity by recognizing CpG specific motifs present in microbial DNA [10]. In the absence of stimulation, TLR9 is retained in the endoplasmic reticulum [10]. Upon activation, TLR9 relocates to the endo-lysosomal compartment, allowing the recruitment of the adaptor molecule MyD88 and stimulation of its subsequent signaling pathways [11]. In dendritic cells, TLR9 is activated after its proteolytic cleavage by asparagine endopeptidase (AEP) and cathepsins [12]. A recruitment and a boost in AEP activity, which was induced shortly after TLR9 activation, has been shown to promote TLR9 cleavage and correlated with an increased acidification in endosomes and lysosomes [12].

The present studies have been undertaken to investigate whether the TLR9 pathway plays a role in host defence against *P. aeruginosa* pulmonary infection. We report that TLR9^-/-^ mice exhibit a significant resistance to lethal infection following pulmonary infection with *P. aeruginosa* compared to wild type (WT) mice. The apparent resistance to *P. aeruginosa* was associated with an improvement of pulmonary bacterial clearance, a better killing of bacteria by AMs and increased productions of IL-1β and nitric oxide (NO), two inflammatory mediators involved in bacteria killing. Taken together these findings suggest that TLR9 down-regulates the innate immune response against *P. aeruginosa*. As a consequence, the absence of TLR9 leads to the improvement of the clearance of *P. aeruginosa* in the lungs and increased mouse survival.

## Materials and Methods

Mice were housed in the Pasteur institute animal facilities accredited by the French Ministry of Agriculture and European regulations (EC Directive 86/609, French Law 2001-486 issued on June 6, 2001). Protocols were approved by the veterinary staff of the Institut Pasteur animal facility (Permit number 04.146) and were performed in compliance with NIH Animal Welfare Insurance #A5476-01 issued on 31/07/2012.

### Reagents

*P. aeruginosa* LPS serotype 10, purchased from Sigma-Aldrich (Saint Quentin Fallavier, France) was purified by gel-filtration chromatography and used at 1 µg/ml. CpG oligonucleotide, 5′-TGA CTG TGA ACG TTC GAG ATG A-3′ purified by HPLC was purchased from Tebu-Bio (Le Perray, France) and used at 1 µg/ml. Bafilomycin A1 and concanamycin B were obtained from Sigma-Aldrich (Saint Quentin Fallavier, France) and used at 10 nM. MV026630 is an acyloxymethyl ketone specific inhibitor of AEP and used at 50 µM as previously decrypted [13]. ODN-2088, the specific TLR9 antagonist and its control, ODN C were purchased from Invivogen (Toulouse, France) and both are used at 1 µg/ml. N-Methyl-L-arginine acetate salts (L-NMMA) was purchased from Sigma-Aldrich (Saint Quentin Fallavier, France) and used at 100 nM. IL-1β receptor antagonist (IL-1βRA) was purchased from Peprotech (Neuilly-Sur-Seine, France) and used at 200 ng/ml.

### Bacterial strain and growth conditions

The wild-type strain PAK, a commonly studied *P. aeruginosa* strain, was obtained from S. Lory (Harvard Medical School, Boston, MA), as originally isolated by D. Bradley (Memorial University of Newfoundland, St. John's, Canada). This strain of *P. aeruginosa* is known to contain and express a full complement of virulence factors, including pili, flagella, the type II secreted enzymes, exotoxin A, elastases and phospholipases and the type III secreted exoenzymes S, T, and Y [14]. The Δ*Pscf* is a PAK mutant with a deletion of *pscF* gene (a major needle protein of the type III secretion system (T3SS), defective in T3SS. PAK and its mutant were prepared as previously reported [6]. Briefly, bacteria were grown overnight in Luria-Bertani broth then transferred to fresh medium and grown for 4–5 h to mid-log phase. The cultures were centrifuged at 4000×g for 15 min. The bacterial pellet was diluted in its original volume and the OD adjusted to give the approximate desired inocula. The inocula were verified by serial 10-fold dilutions of the bacterial suspensions and plating on Luria-Bertani agar.

### Mouse strains

TLR9 deficient mice [15], backcrossed to the C57BL/6 background for 10 generations, were provided by S. Akira (Osaka University, Japan). Mice were fed normal mouse chow and water *ad libitum* and were bred and housed under standard conditions with air filtration.

### Mouse infections and analyses of lung inflammation and bacterial clearance

Mice were anesthetized by i.m. with a mixture of ketamine (40 mg/Kg) and xylazine (8 mg/Kg) and infected intranasally with WT PAK strain at 10^7^ colony-forming unit (CFU) per mouse, as described previously [8]. Briefly, after anesthetization, mice were held by ears and 50 µl of the inoculum ((PAK 10^7^ CFU/mouse) diluted in PBS) were gradually released into the nostrils (25 µl in each nostril) with the help of a micropipette. The rate of release was adjusted to allow mice to inhale the inoculum without trying to form bubbles. Mice were held in the hanging position for another couple of minutes till their breathing gradually returned to normal. The control mice were inoculated intranasally with 50 µl of PBS. Survival experiments, collection of bronchoalveolar lavage fluids (BALs) and cell counts in BALs were performed as previously described [8].

### Culture of mouse AMs and MHS cell line

Mouse primary AMs were isolated as described previously in our laboratory [9]. Briefly, primary cells were isolated from mice after lung washing with PBS. Cells were plated in complete RPMI medium supplemented with 1% sodium pyruvate, 200 mM L-glutamine, 10% (v/v) fetal calf serum, 100 UI/ml penicillin, 100 µg/ml streptomycin, 2.5 mg/l glucose and buffered with 25 mM HEPES. After 2 h, medium was removed and cells were incubated overnight with fresh medium. Mouse AM cell line MHS (CRL-2019; ATCC) was plated in the same complete medium RPMI. Cells were stimulated with CpG 1 µg/ml, or *P. aeruginosa* DNA 25 µg/ml, genomic DNA from lung (eDNA) 25 µg/ml or LPS 1 µg/ml for 24 hours. Primary AMs were infected with PAK, 1 MOI in free medium without serum and antibiotics for 2 or 4 hours. After infection, cells were centrifuged (80 g, 4 min, 4°C) to increase the adherence between cells and bacteria. For the time 24 hours, the cells were infected with PAK, 5 MOI for one hour and then the bacteria were removed and the cells were incubated for 24 hours in culture-free medium. Conditioned media (200 µL) were collected after 2, 4 or 24 hours of incubation, centrifuged (400 g, 5 min, 4°C) to remove bacteria and stored at −20°C.

### Pseudomonas aeruginosa DNA isolation

DNA was extracted from PAK strain using QIAGEN Genomic-tip 500 (Qiagen, Courtaboeuf, France). To remove contaminating lipopolysaccharide (LPS), the DNA was passed through a Detoxi-Gel Endotoxin Removing Resin (Thermo Scientific, Rockford, USA), resulting in DNA preparations with low level of LPS (<1 pg/µg DNA). LPS levels were measured using the Limulus amebocyte lysate kit (Lonza, Basel, Switzerland). The *P.a.* DNA was used at 25 µg/ml. The genomic DNA was extracted from lung and used at 25 µg/ml as a control.

### Histological Studies

Mice were infected with 1×10^7^ CFU of PAK strain and euthanized with pentobarbital 17 hours post-infection. The lungs were then fixed in formol for 48 h, sectioned, and stained with hematoxylin-eosin.

### Analyses of bacterial clearance by AMs

The bacterial clearance was performed as previously described in our laboratory [9]. Briefly, AMs were isolated from mice and infected for 4 h with PAK at MOI of 0.1. CFU were quantified in AMs supernatants and cell lysates pooled together and expressed in percentages using the following formula: (CFU counts recovered without AMs − CFU counts recovered after AM infection) ×100. In certain experiments, AMs were infected for 4 h with a luminescent PAK strain at an MOI 10 and then bacteriolytic activity analysed by measuring the luminescence intensity in supernatants of AMs as detailed below.

### Analyses of bacterial phagocytosis by AMs

Bacterial phagocytosis was performed as previously described in our laboratory [9]. A total of 5×10^5^ AMs were infected with bacteria (MOI = 10) for 1 h. Free and adherent bacteria were removed by washing cells with PBS and were killed with tobramycin treatment (40 µg/mL; 30 min). Then, the cells were washed and lysed in H_2_O containing 0.1% Triton X-100. The number of bacteria in lysates was determined by counting CFU on LB agar plate. The percentage of relative phagocytosis index was assessed as follows: (CFU counts in mutant PAK-treated cells/CFU counts in WT PAK-treated cells) ×100.

### Assays of cytokines and NO production

Murine KC, TNFα, IL-6 and IL-1β concentrations in cell culture supernatants and BAL fluids were determined using DuoSet ELISA assay kits (R&D Systems, Lille, France) with TMB peroxidase substrate (Eurobio, Les Ulis, France). NO production was determined by colorimetric assay based on the Griess reaction [16] using Griess reagent kit (Invitrogen, Saint Aubin, France).

### NF-κB reporter assay

MHS cells were plated at a density of 3×10^4^ cells/ml in 60-mm tissue culture plates 48 hours prior to transfection with an NF-κB reporter plasmid. The reporter plasmid consisted of a luciferase encoding gene controlled by an NF-κB-responsive promoter sequence. Transfection was accomplished by the use of Gene juice from Merck-Millipore (Saint-Quentin-en-Yvelines, France). Transfected cells were plated at 10^5^/well in RPMI with 10% of SVF and incubated for 24 hours before stimulation. The cells were then stimulated with LPS, CpG or LPS/CpG for 8 hours. NF-κB transcriptional activity was assessed by measuring the luciferase activity of cellular lysates and normalized to lysate protein contents. Luciferase activity was quantified by measuring chemiluminescence in cell lysates using luciferase assay system from Promega according to the manufacturer's specifications.

### Western Blot

After incubation, MHS cells were lysed in buffer [5 mM EDTA, 150 mM NaCl, 1% Triton X-100, 50 mM Tris HCl (pH 7.4)] with anti-proteases (Roche Diagnostics). 20 µg of protein lysates were run in a 12% SDS/PAGE, transferred onto PVDF membranes, and probed with antibodies directed against mouse ERK1/2 or its phoshorylated form (Santa Cruz Biotechnology).

### Real-time PCR

RT-PCR was performed using an ABI 7900 RT-PCR detection system (Applied Biosystems, Foster City, CA) in 10 µl reactions that contained 1 µl of diluted cDNA, 300 nM each of forward and reverse primer, and SYBR Green PCR Master Mix (Fisher scientific, Illkirch, France). The primer for murine HPRT has been described in our recent study [17]. Other primers were designed using the Oligo Explorer 1.1. 2 software, murine TLR2 (Fw:5′-GTTTCTGATGGTGAAGGTTG-3′; Rv:5′-GCTGAAGAGGACTGTTATGG-3′), murine TLR4 (Fw:5′- AAATGCCAGGATGATGC-3′; Rw:5′-AGGGACTTTGCTGAGTTTC-3′), murine TLR5 (Fw:5′- TTCAGACGGCAGGATAG -3′; Rw:5′-AAGATTGGGCAGGTTTC-3′), murine TLR7 (Fw:5′-CCACAGGCTCACCCATACTTC-3′; Rv:5′-GGGATGTCCTAGGTGGTGACA-3′), murine TLR9 (Fw:5′-GCACAGGAGCGGTGAAGGT-3′; Rw:5′-GCAGGGGTGCTCAGTGGAG-3′), murine iNOS (Fw:5′- CCC TCC TGA TCT TGTGTT GGA -3′; Rv:5′- CAA CCC GAG CTC CTG GAA -3′).

### Luminescence Activity

PAKLux growth was evaluated using an EGNG Berthold luminometer. As a positive control of bactericidal activity, PAKLux was cultured with tobramycin (40 µg/mL), and the resultant was presented as % of growth vs. PAKLux at T_0_.

### Statistical analyses

Data were represented as means ± SEM. and compared using the unpaired Student's *t* test for the experiments with only two groups. For the experiments with three groups or more, one way ANOVA test was used followed by Bonferroni as a secondary test. For survival curve log-lank (Mantel-cox) test was used to evaluate the significance between groups. *P* values less than 0.05 are considered significant.

## Results

### *P. aeruginosa* DNA stimulates TLR9 in AMs and requires endosomal acidic pH and AEP activation

**Figure 1:**
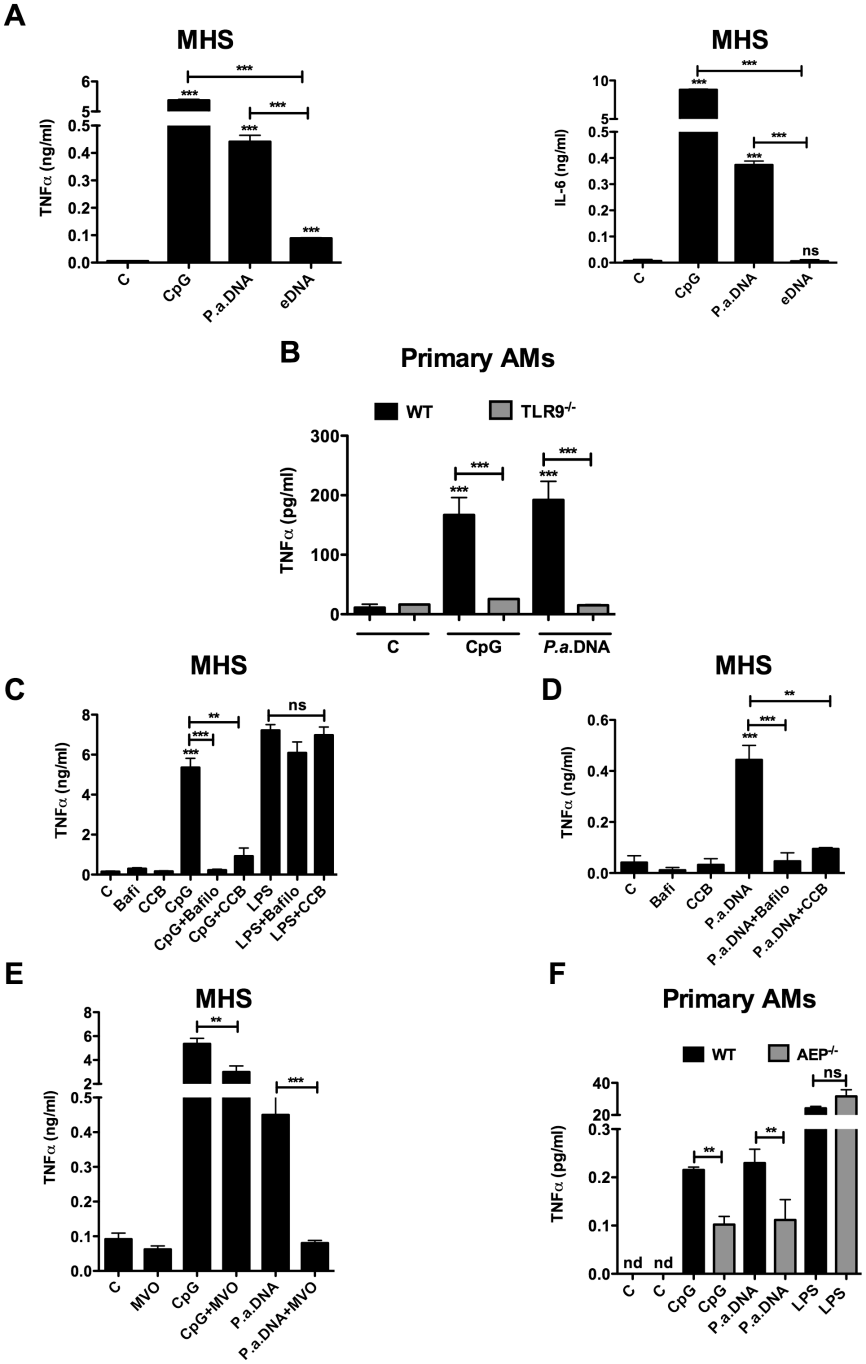
Stimulation of AMs with *P.a*. DNA requires endosomal acidic pH and AEP activation. (**A**) Show the levels of TNFα (*left panel*) and IL-6 (*right panel*) secreted by MHS unstimulated (Control, C) or stimulated with either *P.a*. DNA, *e*DNA (negative control) or with CpG (positive control) for 24 hours. (**B**) Shows TNFα levels produced by WT or TLR9^-/-^ AMs stimulated with *P.a*. DNA, CpG for 24 hours. (**C** and **D**) Show TNFα levels produced by MHS pre-treated for one hour with Bafilomycin A (*Bafilo*) or concanamycin B (*CCB*) and stimulated with *P.a*. DNA, CpG, or LPS. (**E**) Shows TNFα levels produced by MHS pre-treated with the AEP inhibitor MV026630 (MVO) 1 hour and stimulated with CpG or *P.a*. DNA. (**F**) Shows TNFα levels produced by WT and AEP^-/-^ AMs stimulated with CpG, *P.a*. DNA or LPS for 24 hours. Data represent means ± SEM and are representative of three independent experiments *** *P*<0,001 vs C (control), ** *P*<0,01 ns  =  not significant.

We first assessed whether DNA isolated from *P. aeruginosa* (*P.a.* DNA) induces innate immune response through TLR9 signalling pathway in both AMs cell line (MHS) and primary AMs. Our results showed that *P.a*. DNA as well as CpG (the positive control) stimulated TNFα and IL-6 production by MHS (**Fig. 1A**). As a negative control, endogenous DNA (eDNA) extracted from mouse lung had no effect on cytokines production (**Fig. 1A**). Significant levels of TNFα, induced by *P.a*. DNA and CpG, were also detected in the supernatants of AMs derived from WT mice, but were abrogated in AMs from TLR9^-/-^ mice (**Fig. 1B**). Both concanamycin B and bafilomycin A, which increase endosomal pH, inhibited TNFα production triggered by *P.a*. DNA and CpG in MHS (**Fig. 1C, 1D**), suggesting that TLR9 stimulation requires endosomal acidic pH. As a control of specificity, neither concanamycin B nor bafilomycin A compounds had an effect on LPS-induced TNFα production (**Fig. 1C**). In addition, MV026630, an AEP inhibitor, reduced *P.a*. DNA- and CpG-induced TNFα production by MHS (**Fig. 1E**). We verified that MV026630 inhibited AEP activity in these cells (**Fig. S1**). The dependence of TLR9 on AEP activation was also confirmed using AMs from AEP^-/-^ mice. TNFα production induced by *P.a*. DNA was significantly reduced in AMs derived from AEP^-/-^ in comparison to AMs from WT mice (**Fig.1F**). No effect of AEP on TNFα production following stimulation with LPS was observed (**Fig. 1F**). These results suggest that activation of TLR9 by *P. aeruginosa* DNA in AMs requires endosomal acidic pH and AEP activation.

### TLR9 deficiency is associated with increased mouse survival and bacterial clearance during pulmonary infection with *P. aeruginosa*

**Figure 2:**
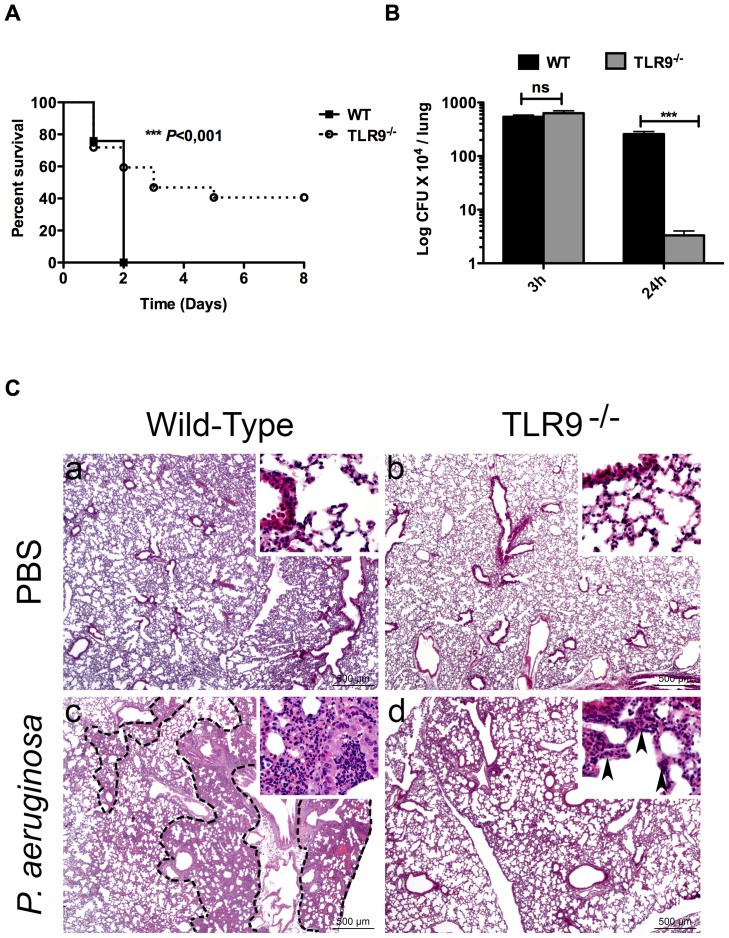
Resistance of TLR9^-/-^ mice to lethal lung infection by *P. aeruginosa*. (**A**) groups of WT and TLR9^-/-^ female mice (n = 10 in each group), were inoculated intranasally with *P. aeruginosa* (PAK strain) at 10^7^ cfu/mouse. Animal survival was determined up to 8 days post-infection. All WT mice died 2 days following lung infection with PAK. Near 40% of TLR9^-/-^ mice resisted to death up to 8 days post infection. (**B**) 3 and 24 hours post-infection, the bacterial load was determined in lungs from both WT and TLR9^-/-^mice. (**C**), Histopathological analysis revealed different lesion profiles between WT and TLR9^-/-^ mice. Control PBS lungs do not display any histological lesion in both WT and TLR9^-/-^ mice (**a,b**). In contrast, 17 hours post-infection with *P. aeruginosa*, (**c**) WT mice displayed a multifocal to coalescing well-delineated inflammatory lesion (dotted line), characterised by an infiltration of neutrophils and macrophages resulting in a complete loss of alveolar spaces and a filling of bronchiolar and alveolar spaces by inflammatory cells and cell debris (inset). (**d**) TLR9^-/-^ mice also displayed an inflammatory lesion characterised by an infiltration of neutrophils and macrophages, but not well-delineated, and sparing the general alveolar structure (inset, black arrowheads). Data ± SEM and are representative of three independent experiments *** *P*<0,001; ns: not significant.

We next examined the role of TLR9 in host defence against pulmonary *P. aeruginosa* infection. We infected WT and TLR9^-/-^ mice (*n* = 10) intranasally with a lethal dose of PAK strain of *P. aeruginosa* and then monitored animal survival for 8 days. As expected, 100% mortality in WT mice was observed within two days post-infection (**Fig. 2A**). However, unexpectedly, 40% of mice lacking TLR9 resisted to death up to 8 days post-infection (**Fig. 2A**). We next examined whether the difference in survival between WT and TLR9^-/-^ mice was due to differences in bacterial clearance in the airways. At the early time point (3 hours) post-infection the same load of bacteria was found in WT and TLR9^-/-^ mice lung (**Fig. 2B**). However, at 24 hours post-infection, the bacterial load was significantly decreased in TLR9^-/-^ compared to WT mice (**Fig. 2B**). This suggests that the increased survival of TLR9^-/-^ mice was likely due to increased bacterial clearance in their lungs. This increase was not associated to changes in the pulmonary expression of TLR2, TLR4 or TLR5 in TLR9^-/-^ mice (**Fig. S2**). Indeed, no significant difference was observed in the levels of TLR2, TLR4 and TLR5 mRNA expression in both lungs and AMs isolated from WT vs. TLR9^-/-^ mice (**Fig. S2A and B**). TLR7 expression was significantly increased in TLR9^-/-^ compared to WT lung, but expression of this receptor had no effect on mouse mortality by *P. aeruginosa* pulmonary infection (**Fig. S2A, C**).

Histopathological analysis revealed no histological lesion in the lung of WT and TLR9^-/-^ instilled with PBS (**Fig. 2C** **a,b**). In contrast, 17 hours post-infection with *P. aeruginosa*, different lesion profiles were identified between WT and TLR9^-/-^ mice. Most WT mice displayed a multifocal to coalescing well-delineated inflammatory lesion, centred on bronchioles and secondary extending to alveoli, leading to a complete loss of alveolar spaces, filled by neutrophils and macrophages (**Fig. 2C** **c**). These lesions coincided with deaths of WT mice that began as early as day 1 post-infection. However, most TLR9^-/-^ mice, displayed a more diffuse and poorly delineated inflammatory lesion, characterised by infiltration of bronchiolar and alveolar walls and spaces by neutrophils and macrophages with preservation of the general alveolar structure (**Fig. 2C** **d**). These results suggest that TLR9 exerts a deleterious effect on mouse survival and airways clearance of *P. aeruginosa*

### TLR9 modulates the host innate responses induced by *P. aeruginosa* pulmonary infection

**Figure 3:**
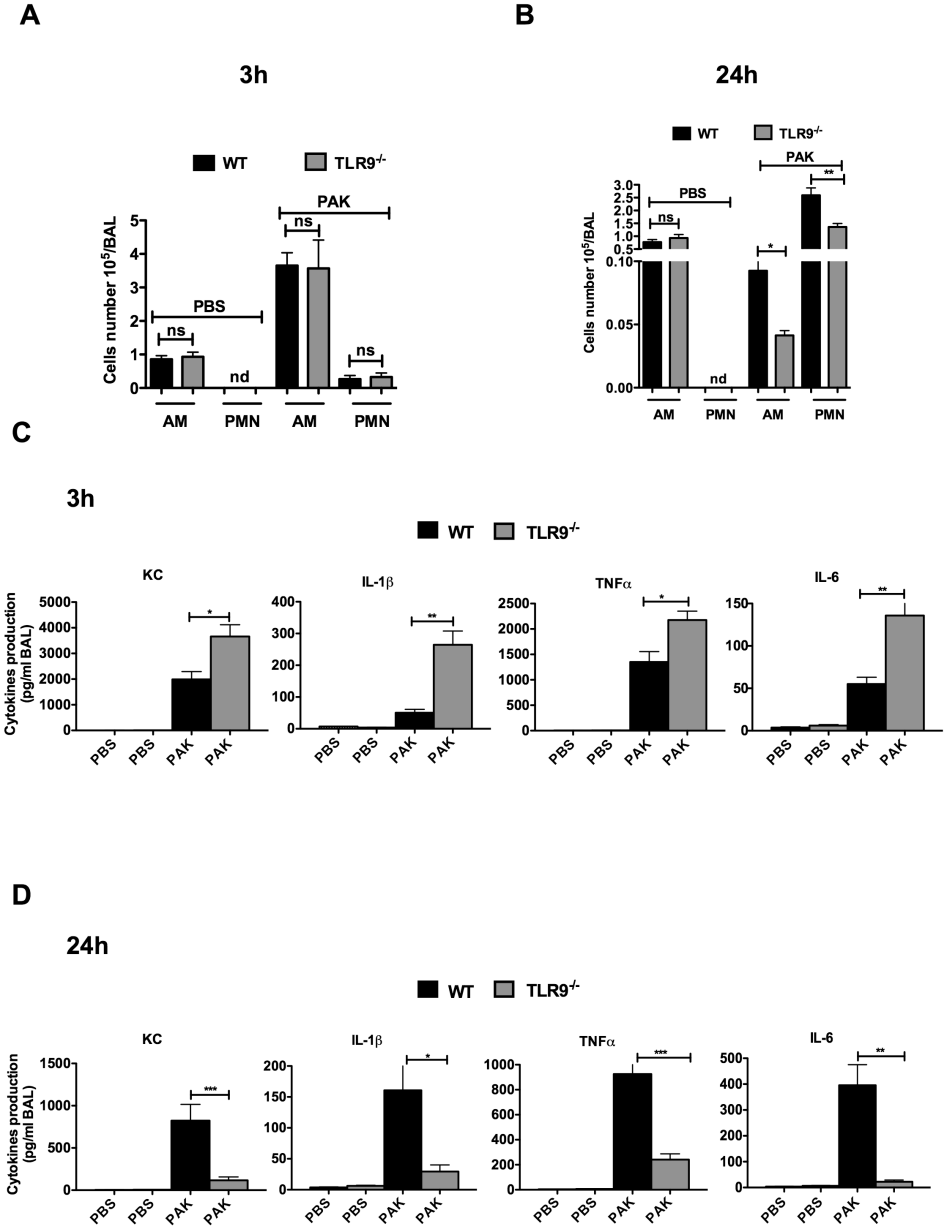
Deletion of TLR9 is associated with an early increase in lung inflammation. BALs from WT and TLR9^-/-^ mice (n = 5 for each group) were collected 3 and 24 hours post-infection. AMs and PMN number recruited by the lung were determined 3 hours (**A**) and 24 hours (**B**) post-infection. KC, TNFα, IL-1β and IL-6 released in BALs were determined 3 hours (**C**) and 24 hours (**D**) post-infection. Data represent means ± SD and are representative of three independent experiments. *** *P*<0,001, ** *P*<0,01, * *P*<0,05. ns  =  not significant, nd  =  not detected.

Given the increased bacterial clearance and resistance to death observed in TLR9^-/-^ mice, we investigated the mechanisms by which TLR9^-/-^ mice control *P. aeruginosa* infection. We first examined lung recruitment of inflammatory cells after *P. aeruginosa* infection. Three hours post-infection, significantly higher numbers of AMs were found in BALs of both WT and TLR9^-/-^ mice compared to PBS-treated mice (**Fig. 3A**). However, no significant differences were observed in AMs number between WT and TLR9^-/-^ mice (**Fig. 3A**). A marked increase in the PMNs number was observed in BALs of WT and TLR9^-/-^ mice at 24 hours after infection. At this time point, both AMs and PMN numbers were significantly lower in TLR9^-/-^ compared to WT mice (**Fig. 3B**). To test whether the innate immune response against *P. aeruginosa* was increased in TLR9^-/-^ mice, we measured inflammatory cytokines levels in BALs. Three hours post-infection, a significant increase in KC, IL-1β, TNFα, and IL-6 levels was detected in BALs from TLR9^-/-^ compared to WT mice (**Fig. 3C**). However, at 24 hours after infection, these levels were detected in BALs of TLR9^-/-^ mice at much lower levels compared to those observed in BALs of WT mice (**Fig. 3D** **)**. These findings suggest that TLR9 modulates the host innate responses induced by *P. aeruginosa* pulmonary infection.

### AMs isolated from TLR9^-/-^ exhibit increased *P. aeruginosa* killing and cytokines production

**Figure 4:**
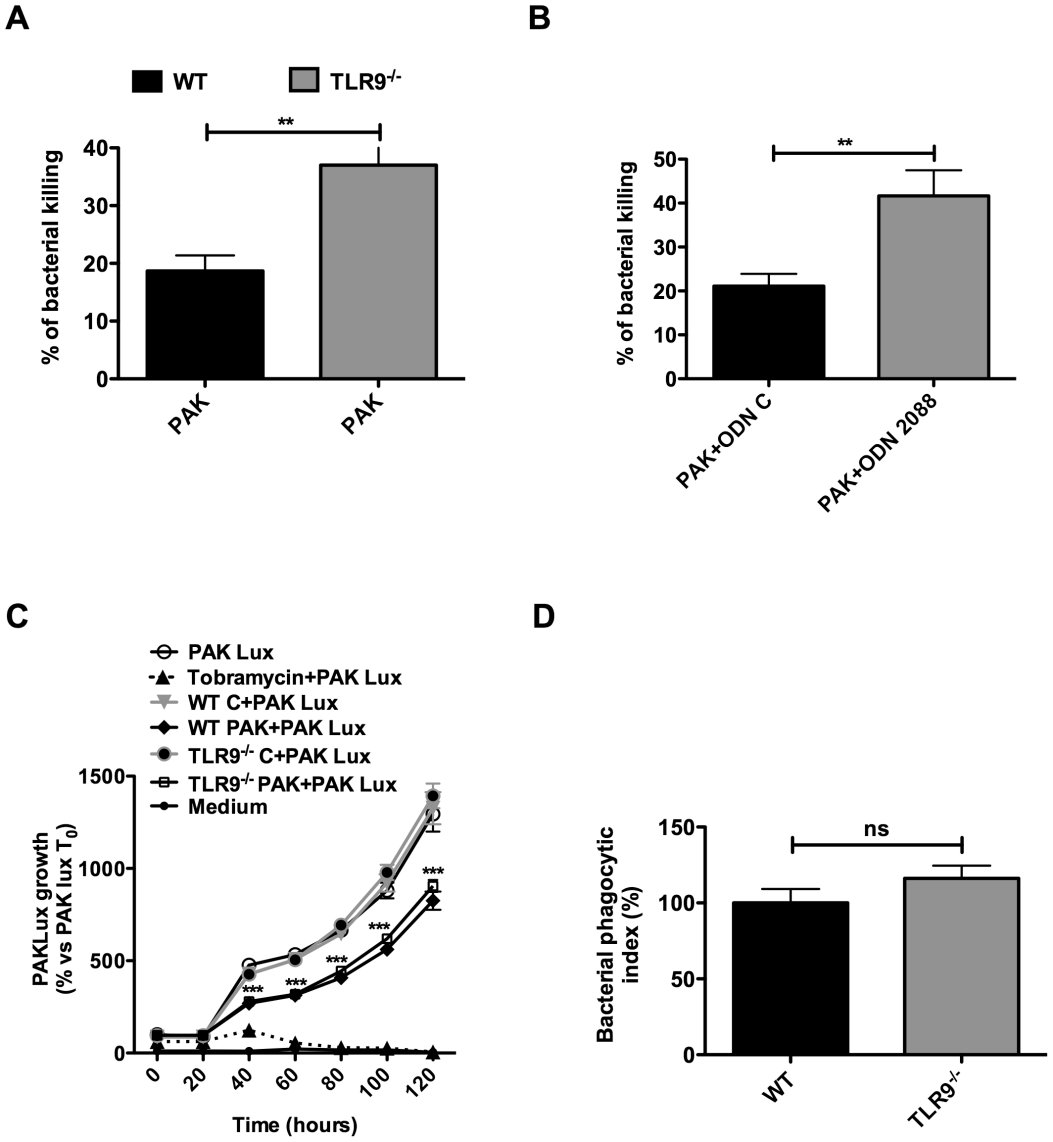
Deletion of TLR9 leads in increased bacterial killing by AMs without interfering with bacterial phagocytosis. (**A and B**) WT and TLR9^-/-^ AMs were infected for 4 hours with PAK (MOI = 0.1) and bacterial killing was quantified as indicated in M&M. **B**) One hour before infection, the cells were stimulated with ODN2088 (1 µg/ml), a specific TLR9 antagonist, or its control ODN (ODN C, 1 µg/ml). (**C**) WT and TLR9^-/-^ AMs were infected with PAK strain (MOI = 1) for 4 hours before collection of cell-free supernatants. The latter were then incubated with a luminescent PAK strain (PAKLux, MOI = 10) followed by killing assay, as indicated in M&M. As a positive control, incubation of PAK-Lux with tobramycin led to a total inhibition of bacterial growth (**D**) bacterial phagocytic activity was measured in AMs of both WT and TLR9^-/-^ mice as indicated in M&M. Results are means ± SEM of three independent experiments. ***P<0.001, **P<0.01, * *P*<0,05.

**Figure 5:**
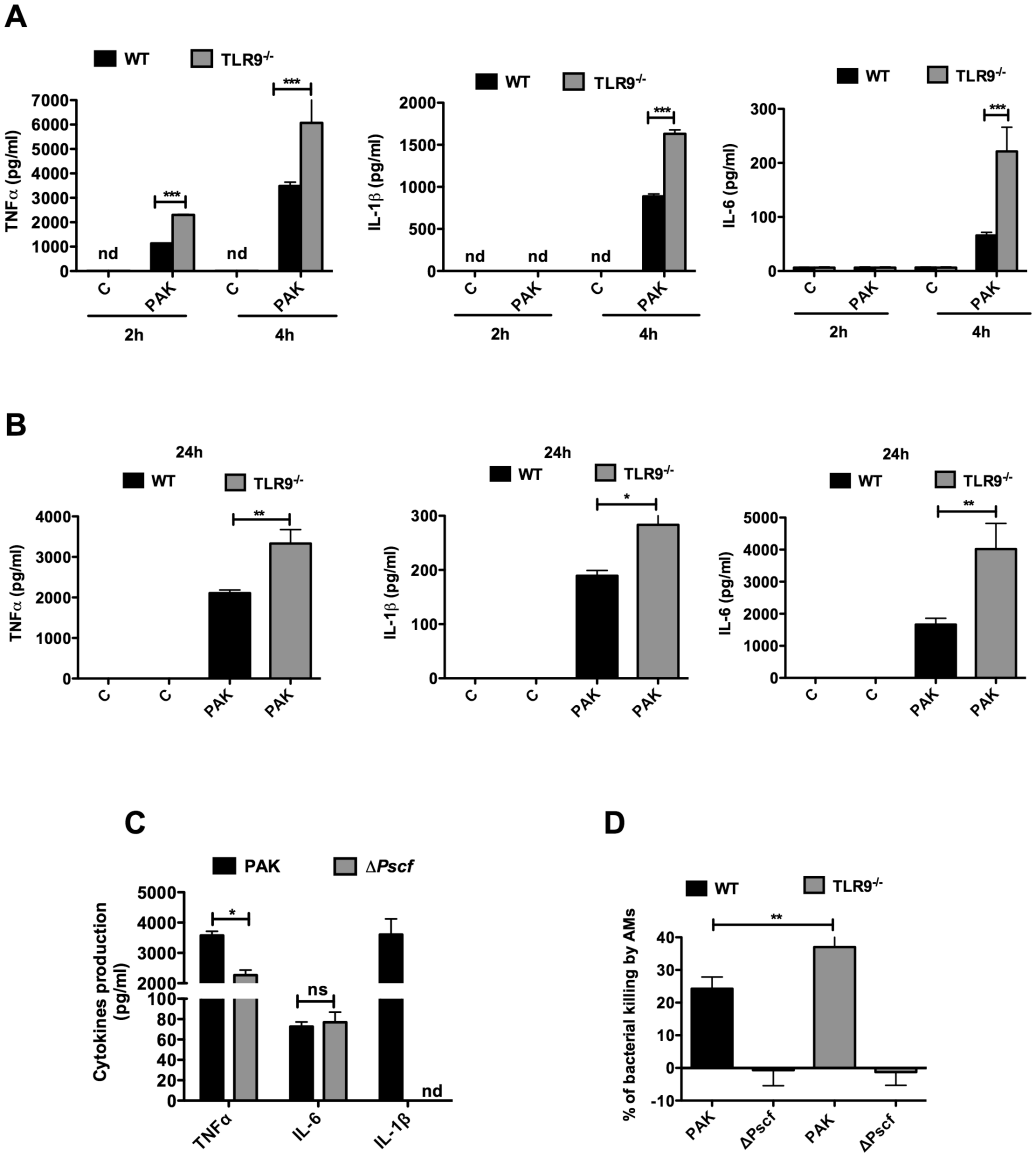
TLR9^-/-^ AMs exhibit increased cytokine secretion following *P. aeruginosa* infection. (**A**) WT and TLR9^-/-^ AMs were infected for 2 or 4 hours with PAK (MOI = 1). (**B**) WT and TLR9^-/-^ AMs were infected for one hour with PAK (MOI = 5) and then the bacteria were removed and the cells were incubated for 24 hours. TNFα, IL-1β and IL-6 levels released in culture supernatants were determined. (**C**) TLR9^-/-^ AMs were infected for 4 hours with PAK or its *ΔPscf* mutant (MOI = 1) and TNFα, IL-1β and IL-6 levels released in culture supernatants were determined. (**D**) Killing assay was performed in WT and TLR9^-/-^ AMs incubated either with PAK or its Δ*Pscf* mutant. Results are means ± SEM of three independent experiments. ***P<0.001, **P<0.01, * *P*<0,05.

In order to determine the mechanisms by which *P. aeruginosa* was cleared more efficiently in TLR9^-/-^ lung, we isolated AMs from both WT and TLR9^-/-^ mice and studied the up-take and bacteria killing by these cells. Interestingly, TLR9^-/-^ AMs infected with *P. aeruginosa* exhibited increased bacteria killing (∼40%) in comparison with WT AMs (∼20%) (**Fig. 4A**). This TLR9-dependent bacterial killing was supported by the fact that the TLR9 specific antagonist, ODN2088, induced a significant increase of bacterial killing in MHS cells (**Fig. 4B**). We then assessed whether this increase of bacteria killing was mediated by a bactericidal extracellular activity, present in cell supernatants, and/or by intracellular killing. Conditioned media collected from non-infected AMs from WT or TLR9^-/-^ mice had no bactericidal effect (**Fig. 4C**). However, media from AMs infected with PAK exhibited increased bactericidal activity without significant difference between WT and TLR9^-/-^ AMs (**Fig. 4C**). In another series of experiments, the bacterial phagocytic test indicated that deletion of TLR9 had no effect on bacterial phagocytosis (**Fig. 4D**). In a subsequent step, we examined the potential involvement of inflammatory cytokines in the increase of *P. aeruginosa* killing by TLR9^-/-^ AMs. A rapid increase of TNFα production was observed in TLR9^-/-^ AMs compared to WT AMs, at both 2 and 4 hours post-infection (**Fig. 5A**). This increase was also observed at 24 hours after infection (**Fig. 5B**). The inflammatory cytokines, IL-6 and IL-1β were not detected at 2 hours post-infection but their levels increased in TLR9^-/-^ AMs, at 4 hours and 24 hours post-infection (**Fig. 5A, B**). As expected, these cytokines were not detected in non-infected cells (**Fig. 5A, B**). Given that IL-1β is known to play a crucial role in intracellular bacterial killing [9], we examined whether this cytokine was associated to the observed increase of bacterial killing in TLR9^-/-^ AMs. We used a PAK mutant (Δ*Pscf*), deficient in the type III secretion system (T3SS), which is unable to induce IL-1β production in WT AMs [9]. TLR9^-/-^ AMs infection with the Δ*Pscf* mutant induced both TNFα and IL-6 but failed to induce IL-1β (**Fig. 5C**). Our result indicates that the Δ*Pscf* mutant was remarkably resistant to killing by both WT and TLR9^-/-^ AMs suggesting that IL-1β was essential for the induction of bacterial clearance (**Fig. 5D**). These data suggest that AMs from TLR9^-/-^ mice exhibit increased inflammatory cytokines production and killing of *P.aeruginosa.*

### IL-1β induced bacteria killing in part through the increase of nitric oxide in lung of TLR9^-/-^ mice

**Figure 6:**
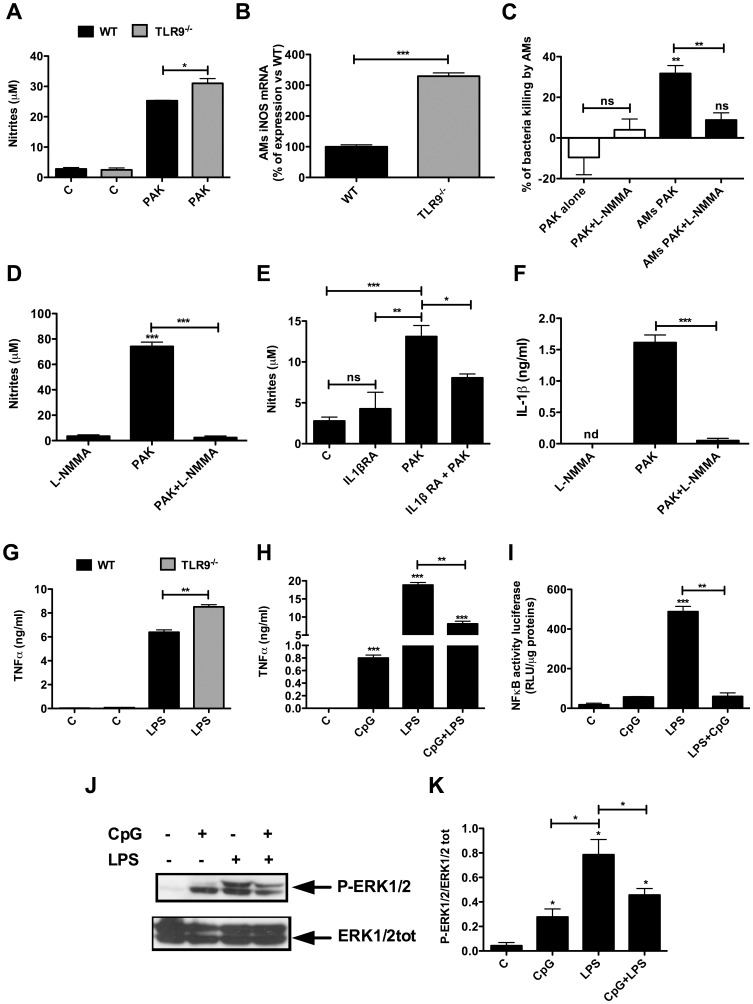
TLR9^-/-^ AMs exhibit an increase in NO production and iNOS expression following *P. aeruginosa* infection. (**A** and **B**) NO production and iNOS mRNA levels were measured in AMs 4 hours post-infection with PAK (MOI = 1). (**C**) Killing assay was performed in WT AMs after inhibition of NO production by L-NMMA (100 nM) one hour before infection for 4 hours with PAK (MOI = 1). (**D**) Nitrite production in WT AMs pre-treated with L-NMMA for 1 hour and infected for 4 hours (MOI = 1) with PAK. (**E**) NO production in WT AMs pre-treated for 1 hour with IL-1βRA (200 ng/ml) before infection for 4 hours with PAK (MOI = 1). (**F**) IL-1β production in WT AMs pre-treated with L-NMMA (100 nM) for one hour before infection for 4 hours with PAK (MOI = 1). (**G**) WT and TLR9^-/-^ AMs were stimulated with LPS (50 ng/ml) for 4 hours. (**H**) MHS cells were stimulated with CpG (1 µg/ml), LPS (1 µg/ml) or CpG+LPS for 24 hours. TNFα levels released in culture supernatants were determined. (**I**) MHS cells were transfected with NF-κB luciferase reporter plasmid for 24 hours and then stimulated with CpG, LPS or CpG+LPS for additional 8 hours, at the same concentrations as in (**H**). The luciferase activity was then measured in cell lysates by luciferase assay. (**J**) MHS cells were stimulated with CpG (1 µg/ml) and/or LPS (1 µg/ml) for one hour. After reaction, total proteins were extracted and ERK1/2 total levels and ERK1/2 phosphorylation were analysed by Western Blotting using specific antibodies. (**K**) shows the quantifications of the blots presented in the figure (**J**). Results are means ± SEM of three independent experiments. *** *P*<0,001, **P<0.01, * *P*<0.05.

Nitric oxide (NO) produced by host immune cells plays a major role in innate immunity due to its ability to kill a broad range of microorganisms [18], [19]. Following infection with *P. aeruginosa*, a significant increase in both NO production and the inducible nitric oxide synthase (iNOS) mRNA was detected in AMs derived from TLR9^-/-^ compared to WT mice (**Fig. 6A, B**). Pharmacological inhibition of iNOS by L-NMMA significantly decreased *P. aeruginosa* killing by AMs (**Fig. 6C**). L-NMMA inhibitor completely abrogated NO production by AMs (**Fig. 6D**). Interestingly, treatment of AMs with IL-1βRA, an antagonist of IL-1β receptor, significantly decreased NO production in AMs following *P. aeruginosa* infection (**Fig. 6E**). Conversely, IL-1β production by AMs was abrogated by L-NMMA (**Fig. 6F**) in parallel to the observed decrease of *P. aeruginosa* killing (**Fig. 6C**). These results indicate that TLR9^-/-^ AMs exhibit increased NO associated to enhanced bacterial killing.

### Stimulation of TLR9 with CpG reduces TLR4 signaling in AMs

The cytokines increase observed in lung of infected TLR9^-/-^ mice and their AMs suggests that TLR9 in our model probably limites the inflammatory response induced by others TLRs like TLR4. LPS is one of the major actors involved in the induction inflammatory response against *P. aeruginosa* in the lung, through the activation of TLR4 [6], [7]. This led us to examine the effect of the TLR9 agonist CpG on LPS-induced AM activation. Our results showed that AMs from TLR9^-/-^ mice produce higher TNFα levels compared to WT AMs under LPS stimulation (**Fig. 6G**). In addition, TLR9 activation with CpG reduced the extent of inflammation induced by LPS in MHS cells through a decrease of NF-κB activation (**Fig. 6H, I**). The increase of NF-κB activation was associated with a significant decrease in ERK1/2 phosphorylation (**Fig. 6J and K**). These results suggest that TLR9 may affect the inflammatory response through inhibition of NF-κB activity.

## Discussion

In the present study we established that TLR9 plays a detrimental role in lung defence against P. aeruginosa. Indeed, we showed that TLR9-/- mice exhibited increased survival and efficient pulmonary clearance of P. aeruginosa in comparison with control mice. This unexpected detrimental effect led us to investigate the underlying cellular and molecular mechanisms by which TLR9 modulated the lung inflammatory response following P. aeruginosa infection. We found that TLR9-/- mice displayed increased levels of airways cytokines at the early stage of P. aeruginosa infection, compared to infected WT mice, leading to the improvement of P. aeruginosa clearance by lungs of TLR9-/- mice. The improvement of airways bacterial clearance resulted in the attenuation of the intensity of late pulmonary inflammation accompanied with reduced airways histological lesions in TLR9-/- mice, 24 h after the initiation of infection.

In an *in vitro* model of murine bone marrow-derived macrophages, TLR9 has been shown to down-regulate the immune response against *Candida albicans* [20]. However, in a mouse model of pneumonia, TLR9 exhibited a protective effect against the Gram-negative bacterium, *Klebsiella pneumonia* [21], [22]. In that study, TLR9^-/-^ mice displayed significantly increased mortality following intra-tracheal infection with *Klebsiella pneumonia* [21], [22]. This was associated to impaired bacterial clearance and activation of type I cytokine production. On the same line, it was demonstrated that TLR9 played a protective role in airway infection with *Streptococcus* pneumonia [23]. Thus, it is likely that the role of TLR9 in the early immune response may vary depending of the animal model and the pathogen used. This difference can be explained by the extent of TLR9 activation depending on the composition of the genomic DNA in different bacteria. The efficient anti-inflammatory role of TLR9 in our model may be due to the high GC content (66,6%) and hence abundance of unmethylated CpG motifs *in P. aeruginosa* DNA [24].

Given that AMs are well known to contribute to the early innate defence in the lungs by phagocytosing pathogens [25], we examined the regulation by TLR9 of the inflammatory response and *P. aeruginosa* killing by these cells. Our results indicated that TLR9 deficiency increased the inflammatory cytokine productions by isolated AMs. This increase was not a transient phenomenon as it was observed at both the early stage (2 and 4 hours) and late stage (24 hours) following infection of these cells by *P. aeruginosa*. This led to an increase in the killing activity of AMs toward *P. aeruginosa* and promoted the production by these cells of IL-1β and NO, two inflammatory mediators involved in bacterial killing. The enhanced bactericidal activity of AMs may explain the improvement of *P*. *aeruginosa* clearance in airways by TLR9^-/-^ mice. The enhanced bacterial clearance was not due to an increased bacterial uptake by TLR9^-/-^ AMs, suggesting that in these cells TLR9 did not modulate the expression of receptors involved in bacterial uptake. The observed increase of cytokines production by AMs of TLR9^-/-^ mice suggested that, in spite of the ability of *P. aeruginosa* DNA to stimulate TLR9, the latter exerted an anti-inflammatory effect when AMs were stimulated by the whole bacterium. It is likely that TLR9 down-regulated the activation of AMs by other PAMPs of *P*. *aeruginosa* such as LPS (the specific ligand of TLR4) and that the removal of TLR9 led to an increased AMs activation by LPS. Inhibition of NF-kB translocation appears to be one of the mechanisms by which TLR9 may impair AM activation by other PAMPs. Indeed, our studies showed that TLR9 down-regulated NF-kB activation by LPS but the mechanisms involved in this down-regulation are still unclear. Our findings showed that CpG abolished induction of TNFα production in AMs stimulated by LPS from P. aeruginosa by interfering with NF-κB and ERK1/2 signalling pathways. It has been shown that TLR9 activation by DNA of various Lactobacillus species inhibited H2O2-induced IκB-alpha degradation and NF-κB translocation to the nucleus [26]. These findings suggest that TLR9 down-regulated inflammatory reaction by inhibiting NF-κB signalling pathways.

The cytokine increase observed at the early stage of lung infection with *P. aeruginosa* in TLR9^-/-^ mice suggested that TLR9 limited the inflammatory response induced by *P. aeruginosa* via other TLRs. Indeed, *P*. *aeruginosa* has been shown to stimulate lung inflammation *via* TLR4 and TLR5-dependent process [6], [7]. In this regard, it was demonstrated that activation of TLR9 with CpG limited TLR4 signalling in enterocytes leading to the reduction of the extent of intestinal inflammation [27], [28]. In addition, it has been shown that the inhibition of TLR9 conferred protection from liver injury in ischemia/reperfusion model [29].

We next examined the relationship between increased bacterial clearance of *P*. *aeruginosa* by AMs of TLR9^-/-^ mice and IL-1β production by these cells. Our results showed that a PAK mutant (Δ*Pscf*), unable to induce IL-1β production by AMs, was remarkably resistant to killing by TLR9^-/-^ AMs, compared to what we previously observed with WT AMs [9]. This finding together with the increased IL-1β production by TLR9^-/-^ AMs confer a key role to IL-1β in the increased ability of these cells to kill *P*. *aeruginosa*. On the other hand, our results showed that *P*. *aeruginosa* induced increased iNOS transcription and NO production in part via an IL-1β-dependent mechanism. This cytokine is known to stimulate NO production through the increase of iNOS mRNA transcription [30]. Microbicidal effect of NO is mediated by a direct interaction of NO with the DNA repair system of pathogens, by damaging their membrane lipids, or by modulating the host immune response [31], [32], [33]. These mechanisms may potentially play a role in the modulation of *P. aeruginosa* killing by TLR9 in AMs. Our findings are in agreement with previous studies showing the implication of IL-1β in the induction of NO production. Indeed, It has been shown that IL-1β induced NO production in murine macrophages [34] and human A549 epithelial cells through the activation of the iNOS expression [35], [36].

In summary, this study reports, for the first time, that TLR9 down-regulates the innate immune response against *P. aeruginosa* and that the absence of TLR9 leads to an early increase in the inflammatory response. As a consequence, this leads to the improvement of the clearance of *P. aeruginosa* in the lungs and increased mouse survival. The apparent enhancement in the ability of TLR9^-/-^ mice to eliminate *P. aeruginosa* seems to be due to the fact that TLR9 deletion improves AMs killing of this bacterium by increasing the production of IL-1β and NO, two inflammatory mediators necessary for *P. aeruginosa* killing by AMs. Our findings would help design future therapeutic strategies, based on TLR9 inhibition, to control *P. aeruginosa*-induced pneumonia.

## Supporting Information

Figure S1 MV026630 inhibits AEP activity in CpG stimulated MHS cells. Cells were incubated with MV026630 50 µM for 1 hour before and during stimulation with CpG 1 µg/ml for 24 hours. AEP activity was measured as indicated in M&M. ** *P*<0,01.
(TIFF)

Figure S2 Comparative levels of TLRs mRNA expression in lungs of WT vs. TLR9^-/-^ mice. (**A, B**) TLR2, −4, −5, −7 and −9 mRNA levels expressed in the lung or AMs from WT vs. TLR9^-/-^ mice was determined, as indicated in M&M. (**C**) WT and TLR7^-/-^ mice (n = 10 in each group) were inoculated intranasally with *P. aeruginosa* 10^7^ CFU. Mice survival was determined for up to 4 days post-infection. Data represent means ± SEM and are representative of three independent experiments *** *P*<0,001; ns: not significant.
(TIFF)
