# Supplementary figures and images for: Toll-Like Receptor 9 Deficiency Protects Mice against Pseudomonas aeruginosa Lung Infection
Source: PLoS One. 2014 Mar 4;9(3):e90466. doi: 10.1371/journal.pone.0090466 (PMC3942450; doi:10.1371/journal.pone.0090466)

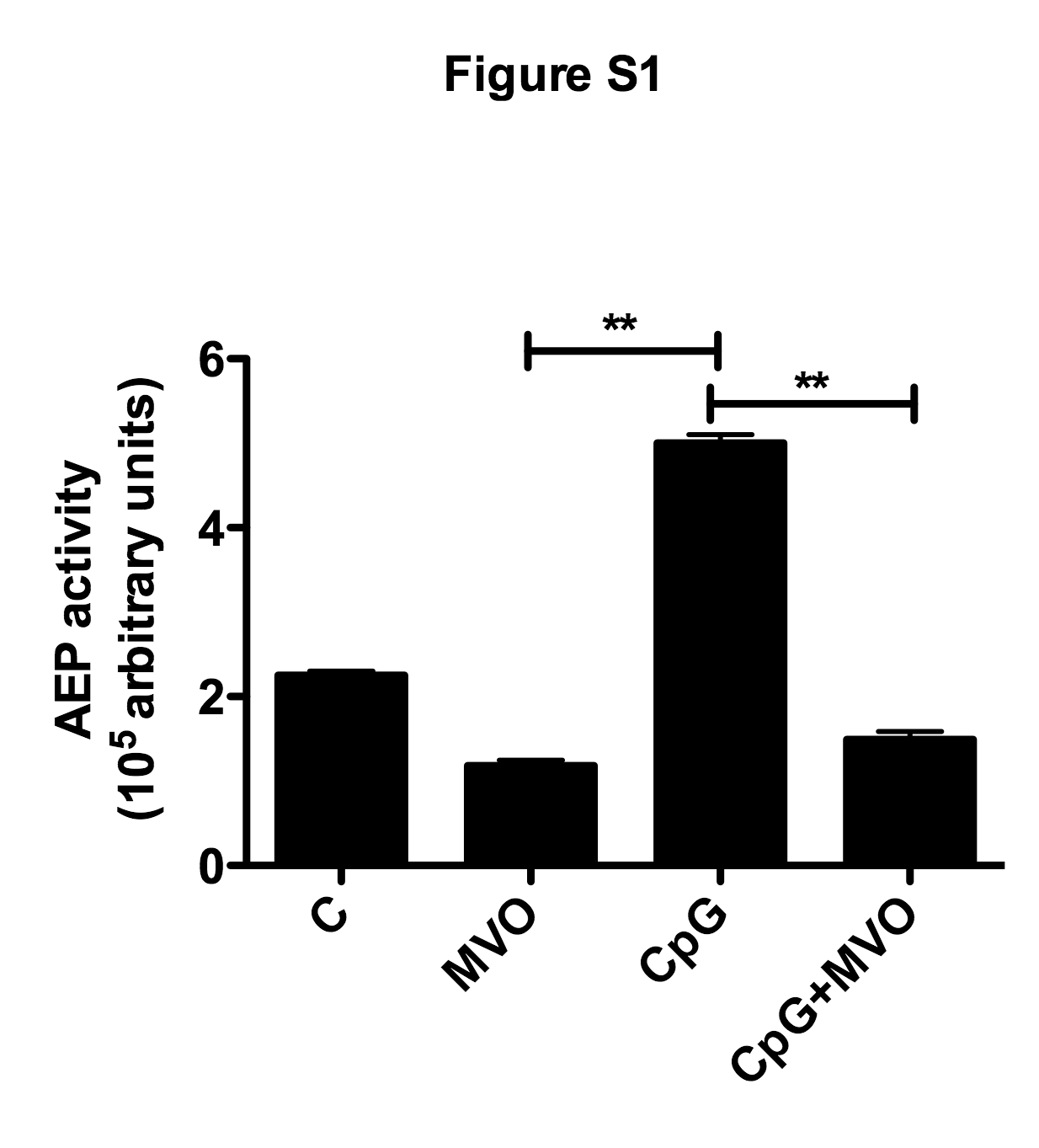

Supplement: Figure S1 — MV026630 inhibits AEP activity in CpG stimulated MHS cells. Cells were incubated with MV026630 50 µM for 1 hour before and during stimulation with CpG 1 µg/ml for 24 hours. AEP activity was measured as indicated in M&M. ** P<0,01. (TIFF) [file pone.0090466.s001.tiff]

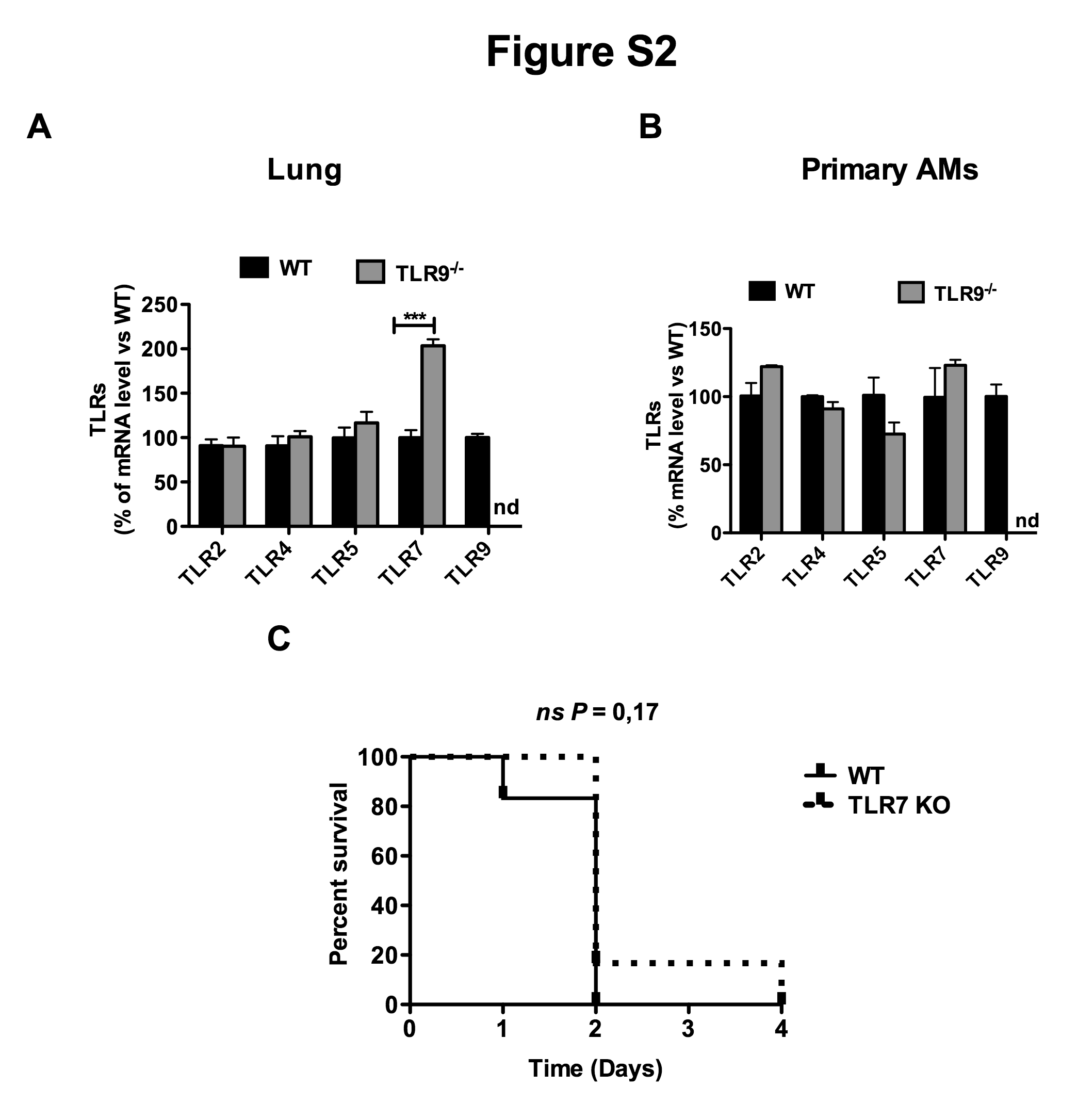

Supplement: Figure S2 — Comparative levels of TLRs mRNA expression in lungs of WT vs. TLR9-/- mice. (A, B) TLR2, −4, −5, −7 and −9 mRNA levels expressed in the lung or AMs from WT vs. TLR9-/- mice was determined, as indicated in M&M. (C) WT and TLR7-/- mice (n = 10 in each group) were inoculated intranasally with P. aeruginosa 107 CFU. Mice survival was determined for up to 4 days post-infection. Data represent means ± SEM and are representative of three independent experiments *** P<0,001; ns: not significant. (TIFF) [file pone.0090466.s002.tiff]
